# Synthetic Mirror Bacteria as a Frontier for Chiral Synthetic Biology and Biocontainment

**DOI:** 10.1002/mbo3.70356

**Published:** 2026-06-23

**Authors:** Mohammad Nazrul Islam Bhuiyan

**Affiliations:** ^1^ BCSIR Chattogram Laboratories, BCSIR Chattogram Bangladesh; ^2^ Institute of Food Science and Technology, Bangladesh Council of Scientific and Industrial Research (BCSIR) Dhaka Bangladesh

**Keywords:** antimicrobial strategy, chiral medicine, mirror bacteria, mirror‐image antibiotics, prophylactic agents, synthetic biology

## Abstract

Chirality is a fundamental structural property of biological molecules that governs molecular recognition, enzymatic catalysis, and genetic information processing in living systems. Natural life exhibits a universal pattern of homochirality in which proteins are composed predominantly of l‐amino acids, while nucleic acids contain d‐sugars within their backbone structures. Advances in synthetic biology and chemical biology have stimulated growing interest in mirror biological systems that operate with inverted molecular chirality. In theory, mirror organisms would contain proteins composed of d‐amino acids and nucleic acids built from l‐sugars, forming a stereochemically inverted yet internally consistent biochemical framework that is largely incompatible with natural biological systems. This review examined the molecular foundations, engineering strategies, biosafety considerations, and ecological implications associated with the theoretical development of mirror bacteria. Particular emphasis is placed on the hierarchical organization of biological chirality and the stereochemical constraints that govern macromolecular folding, molecular recognition, and the processing of genetic information. Recent advances in the chemical synthesis of mirror proteins and mirror nucleic acids demonstrate that stereochemically inverted biomolecules can adopt stable structures and perform catalytic or informational functions. However, integrating these components into self replicating mirror cellular systems remains a major scientific challenge. Furthermore, the ecological interactions, evolutionary dynamics, and environmental persistence of mirror biological systems require careful biosafety evaluation and responsible governance. This review highlights key conceptual and technological challenges that must be addressed before mirror organisms can progress from theoretical constructs toward experimental feasibility.

## Graphical abstract

This work explores mirror (d‐chiral) bacteria as a fundamentally orthogonal form of life, demonstrating their resistance to natural antibiotics and potential for biocontained biotechnology. Mirror cells open new frontiers in antimicrobial design, synthetic biology, ecology, and astrobiology by redefining biochemical compatibility and evolutionary constraints.

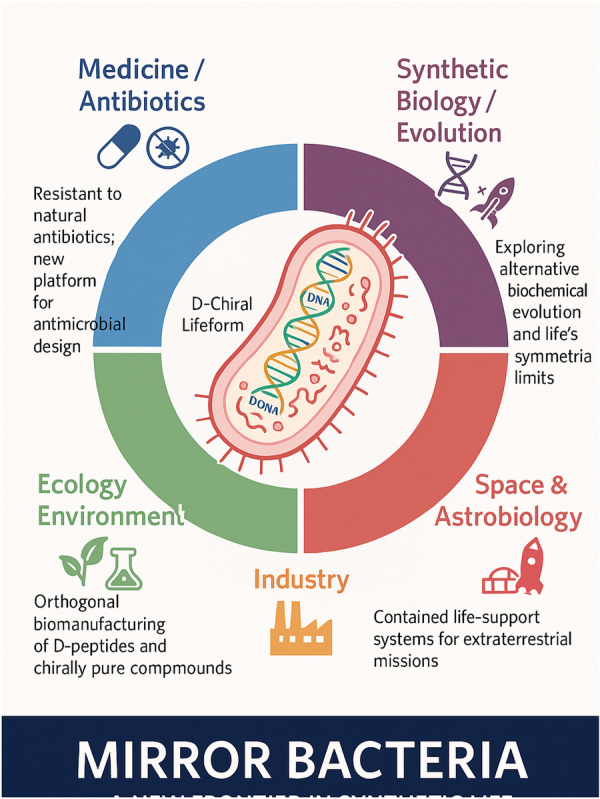

## 1 Introduction

Chirality represents a fundamental structural property of biological molecules in which two configurations exist as non‐superimposable mirror images known as enantiomers. In living systems, this stereochemical asymmetry is expressed as biological homochirality, a universal characteristic whereby proteins are constructed almost exclusively from l‐amino acids while nucleic acids contain d‐ribose or deoxyribose sugars in their phosphate backbones. This asymmetric organization is conserved across all known life forms and is essential for the stability of macromolecular structure, enzyme catalysis, and genetic information transfer (Blackmond 2010; Gonda et al. 2023; Inaki et al. 2016; Lopez and Mohiuddin 2025). Homochirality enables precise molecular recognition among biomolecules, allowing proteins, nucleic acids, and metabolites to interact through highly stereospecific binding interfaces. Even minor deviations from this stereochemical organization can disrupt protein folding, enzymatic activity, and cellular signaling pathways, demonstrating that chirality is deeply embedded in the architecture of biological systems (Cava et al. 2011; Sasabe and Suzuki 2018).

Rather than representing a simple binary property, biological chirality operates as a hierarchical structural constraint that propagates across multiple levels of molecular organization. At the molecular level, chiral centers within amino acids and d‐sugars determine the handedness of secondary structural motifs such as alpha helices and beta sheets. These local geometries further influence tertiary protein folding, supramolecular assembly, and the architecture of macromolecular complexes such as ribosomes and polymerases. At the systems level, homochirality governs metabolic compatibility, enzymatic recognition, and information flow across cellular networks. The evolutionary entrenchment of this stereo‐chemical hierarchy explains why life has converged on a single chiral configuration and why mixed chiral systems are typically unstable or biologically dysfunctional (Blackmond 2010; Kubyshkin and Budisa 2019; Liu 2021). Consequently, attempts to engineer alternative forms of life must address not only the inversion of molecular chirality but also the structural and functional constraints that arise from this hierarchical organization.

The concept of mirror life has emerged from advances in synthetic biology, xenobiology, and chemical genome engineering. Mirror biological systems would reverse the stereochemical configuration of the canonical building blocks of life by constructing proteins from d‐amino acids and nucleic acids from l‐sugars. Such mirror organisms would represent a form of parallel biology that is stereochemically incompatible with natural organisms and therefore functionally orthogonal to the existing biosphere (Budisa et al. 2020; Schmidt 2010). Because molecular recognition processes in biology are highly stereospecific, natural enzymes, antibodies, and viruses generally fail to recognize mirror biomolecules. This property has motivated growing interest in mirror biochemistry as a potential strategy for achieving intrinsic biological containment and expanding the chemical space accessible to engineered biological systems (Acevedo‐Rocha and Budisa 2011; Karbalaei‐Heidari and Budisa 2024).

Progress toward mirror life has primarily occurred at the level of individual biomolecules rather than whole cells. Experimental studies have demonstrated the chemical synthesis of mirror image proteins, enzymes, and nucleic acid systems capable of performing catalytic or informational functions. For example, mirror enzymes synthesized from d‐amino acids can adopt stable folded conformations and retain catalytic activity, demonstrating that protein folding principles are preserved across enantiomeric systems (Weinstock et al. 2014; Harrison et al. 2023). Similarly, advances in mirror nucleic acid chemistry have enabled mirror replication and transcription processes using synthetic molecular systems, illustrating the feasibility of mirror genetic information transfer (Wang et al. 2016). Additional progress has been achieved through mirror image phage display and mirror protein sequencing technologies, which provide powerful platforms for the discovery and characterization of d‐protein therapeutics (Qi et al. 2024; Zhang and Zhu 2024). Despite these advances, the integration of mirror macromolecules into a self‐sustaining cellular system remains a formidable challenge.

A central obstacle in the construction of mirror organisms arises from stereochemical incompatibility between molecular components of opposite chirality. Mixed chiral systems cannot easily maintain uniform helical geometry in nucleic acids, consistent folding patterns in proteins, or reliable catalytic recognition between enzymes and substrates. These incompatibilities extend to essential biological processes, including DNA replication, transcription, translation, and metabolism. Consequently, mirror life would require the parallel synthesis of an entirely mirror biochemical infrastructure that includes mirror ribosomes, mirror polymerases, mirror metabolic enzymes, and mirror membrane biosynthesis pathways. Recent work in orthogonal translation systems and expanded genetic codes provides partial solutions to these challenges by enabling the incorporation of non‐canonical‐amino acids and the development of synthetic translation machinery (Liu and Schultz 2010; Wang et al. 2009; Liu et al. 2017). However, constructing a fully functional mirror central dogma that operates independently of natural biochemical systems remains an unresolved frontier.

Beyond molecular engineering challenges, mirror biology also intersects with broader questions concerning the origin and diversity of life. Theoretical models such as the alanine world hypothesis propose that the evolutionary selection of specific amino acid stereochemistries played a crucial role in shaping the structural landscape of early proteins and metabolic pathways (Kubyshkin and Budisa 2019). Similarly, research on foldamers has demonstrated that the stereochemical configuration of monomers strongly constrains the folding space available to macromolecules, thereby influencing catalytic capability and structural stability (Gellman 1998). These insights highlight that the feasibility of mirror biological systems cannot be evaluated solely through molecular inversion but must consider the broader structural principles that govern biomolecular organization and evolution.

Interest in mirror biology is also driven by potential applications in medicine, biotechnology, and biosafety. Mirror peptides and d‐protein therapeutics are inherently resistant to degradation by natural proteases and therefore exhibit improved pharmacokinetic stability compared with conventional peptide drugs (Lander et al. 2023). Recent studies have demonstrated that mirror image binding proteins and monobodies can selectively target disease associated proteins, including oncogenic kinases, providing promising avenues for therapeutic development (Schmidt et al. 2024). In addition, xenobiological organisms constructed with alternative genetic systems have been proposed as intrinsically contained microbial platforms that reduce the risk of horizontal gene transfer or uncontrolled environmental proliferation (Acevedo‐Rocha and Budisa 2011; Schmidt 2010). Such orthogonal biological systems may therefore contribute to the development of safer synthetic biology technologies.

Despite these potential benefits, the prospect of creating mirror organisms has raised significant biosafety and ethical concerns. A multidisciplinary analysis involving synthetic biology, immunology, and ecological science has emphasized that mirror life could pose ecological risks if introduced into natural environments (Adamala et al. 2024). Because mirror organisms would not be recognized by natural viruses or many biological predators, their ecological interactions may differ substantially from those of conventional microbes. Furthermore, uncertainties remain regarding the metabolic capabilities of mirror cells, their interactions with natural ecosystems, and the long term evolutionary dynamics of stereochemically isolated life forms. These considerations have led researchers to emphasize that the development of mirror organisms must proceed cautiously and should prioritize robust biocontainment strategies and international governance frameworks (Li et al. 2026; Karbalaei‐Heidari and Budisa 2024).

Although complete mirror cells have not yet been constructed, rapid progress in genome synthesis, synthetic translation systems, and orthogonal biochemical networks has brought the concept of mirror biology closer to experimental realization. The chemical synthesis and rewriting of bacterial genomes has already demonstrated the capacity to redesign genetic systems at the whole genome scale (Venetz et al. 2019). Advances in synthetic ecosystems and engineered microbial consortia further illustrate how synthetic biology can create novel biological interactions and metabolic networks that do not exist in nature (Solé et al. 2024; Jiang et al. 2025). These developments provide conceptual and technical foundations that may eventually enable the construction of mirror biological systems.

This review examines the emerging field of mirror bacteria within the broader context of chiral synthetic biology and xenobiology. The objective is to critically evaluate the biochemical principles that govern stereochemical organization in living systems and to assess the feasibility of constructing mirror cellular life. Particular attention is given to stereochemical constraints, foldamer based structural considerations, orthogonal translation systems, and experimental biocontainment strategies. By integrating insights from chemical biology, synthetic genomics, and systems biology, this review aims to clarify both the scientific opportunities and the potential risks associated with mirror biological systems. Understanding these dimensions is essential for guiding responsible innovation in the rapidly evolving landscape of synthetic life engineering.

## 2 Molecular Foundations of Mirror Biology and Chiral Hierarchy

**Figure 1:**
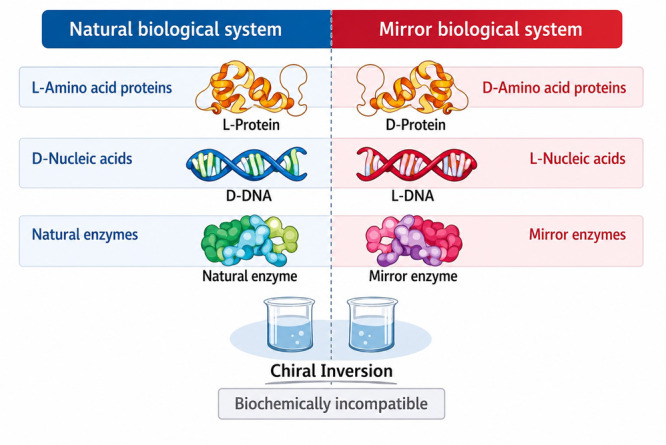
Conceptual comparison of natural biological systems and mirror biological systems.

The feasibility of mirror biological systems cannot be understood solely through the inversion of molecular chirality. Biological chirality operates as a multilevel structural principle that constrains molecular recognition, macromolecular folding, and cellular organization. In natural organisms this stereochemical organization manifests as biological homochirality, where proteins are composed of l‐amino acids and nucleic acids incorporate d‐ribose sugars (Figure 1). This configuration enables the consistent geometry required for enzyme catalysis, nucleic acid replication, and molecular recognition within cellular networks (Blackmond 2010; Gonda et al. 2023).

Homochirality is maintained through hierarchical interactions across several levels of biological organization. At the molecular level, the configuration of chiral centers in amino acids determines the handedness of secondary structures such as alpha helices and beta sheets. These structural motifs influence tertiary protein folding and the formation of catalytic active sites. At the supramolecular level, the stereochemical orientation of macromolecules governs interactions among proteins, nucleic acids, membranes, and metabolites. Ultimately, this hierarchy of stereochemical compatibility enables coordinated metabolic networks and reliable genetic information processing. Disruption of this hierarchical organization often leads to misfolded proteins, loss of catalytic activity, or impaired molecular recognition (Sasabe and Suzuki 2018).

Mirror biology proposes the construction of an alternative stereochemical framework in which this hierarchy is inverted but internally consistent. In such systems proteins would be composed of d‐amino acids, while nucleic acids would contain l‐sugars. This configuration would produce a biochemical architecture that mirrors natural life but remains incompatible with it. Because enzymatic recognition is highly stereospecific, natural enzymes, polymerases, and ribosomes generally cannot process mirror substrates. Consequently mirror biological systems would operate independently from natural biochemical pathways and form a stereochemically isolated biosystem (Budisa et al. 2020; Schmidt 2010).

A critical concept in evaluating mirror biology is stereochemical closure. Biological processes such as DNA replication, transcription, translation, and metabolism depend on coordinated interactions between macromolecules of compatible chirality. If a single component of this system possesses opposite chirality, molecular recognition fails and the process becomes inefficient or impossible. For example, polymerases cannot efficiently replicate nucleic acids with opposite handedness because the helical geometry and catalytic interfaces are incompatible. Similarly ribosomes evolved to synthesize l‐proteins exhibit limited ability to incorporate d‐amino acids during translation (Katoh et al. 2017). These constraints demonstrate that mirror organisms cannot be produced simply by reversing one molecular component but require reconstruction of an entire mirror central dogma.

Insights from foldamer chemistry further illustrate how monomer stereochemistry constrains macromolecular structure and function. Foldamers are synthetic oligomers that adopt stable folded conformations determined by the stereochemical properties of their building blocks. Research in this field demonstrates that stereochemistry strongly influences folding pathways, structural stability, and catalytic capability (Gellman 1998). These principles also apply to biological proteins, where the stereochemistry of amino acids determines accessible folding landscapes. The alanine world model proposes that early evolutionary selection of amino acid stereochemistry shaped the structural possibilities of proteins and metabolic systems (Kubyshkin and Budisa 2019). Understanding these structural constraints is essential for evaluating the feasibility of mirror biological systems.

Advances in synthetic biology have begun to explore these possibilities through the construction of mirror biomolecules and orthogonal translation systems. Experimental work has demonstrated that mirror proteins composed of d‐amino acids can adopt folded structures and perform catalytic functions similar to natural enzymes (Harrison et al. 2023; Weinstock et al. 2014). Parallel progress has occurred in mirror nucleic acid chemistry, where l‐DNA and l‐RNA molecules have been synthesized and shown to support mirror genetic information transfer (Wang et al. 2016). These studies demonstrate that the fundamental chemical principles underlying biological macromolecules remain valid when stereochemistry is inverted.

Despite these advances, the construction of mirror organisms remains a formidable challenge because all major cellular systems must operate within the same stereochemical framework. Mirror DNA replication requires mirror polymerases, mirror transcription systems require mirror RNA polymerases, and mirror translation requires ribosomes capable of synthesizing d‐proteins. Furthermore, metabolic networks must be reconstructed using mirror enzymes capable of catalyzing biochemical reactions with mirror substrates. The engineering of such integrated systems represents one of the most ambitious challenges in synthetic biology and chemical biology.

Understanding the molecular foundations of mirror biology therefore requires integrating insights from stereochemistry, structural biology, synthetic genomics, and systems biology. By examining how chirality constrains molecular interactions across multiple levels of biological organization, researchers can better evaluate both the feasibility and limitations of mirror life. This framework provides the conceptual basis for the subsequent sections of this review, which examine technological strategies, potential applications, and biosafety considerations associated with mirror biological systems.

## 3 Chirality Hierarchy and Stereochemical Constraints in Mirror Life

Chirality represents one of the most fundamental organizing principles in biological systems. Rather than functioning as a simple molecular attribute, chirality operates through a hierarchical framework that governs structural organization from the level of atomic stereocenters to entire cellular networks. In natural life, this hierarchy is manifested through biological homochirality, where proteins are constructed from l‐amino acids while nucleic acids incorporate d‐sugars. This consistent stereochemical configuration ensures the geometric compatibility required for macromolecular folding, enzymatic catalysis, and accurate genetic information processing (Blackmond 2010; Gonda et al. 2023). The persistence of homochirality across all known life forms suggests that it is deeply embedded in the evolutionary architecture of biological systems.

### 3.1 Hierarchical Organization of Biological Chirality

**Figure 2:**
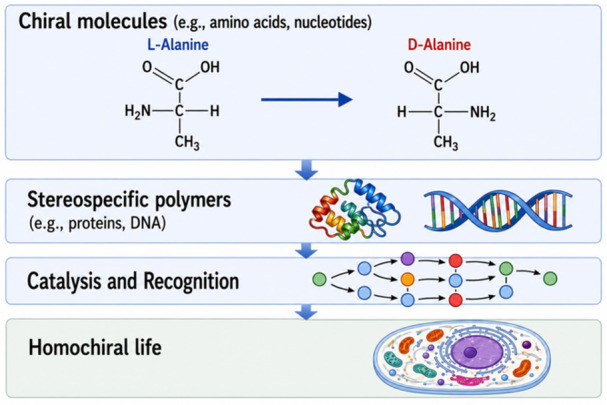
Hierarchical propagation of chirality in biological systems.

The hierarchical organization of biological chirality spans multiple structural levels, beginning with the stereochemical properties of individual biomolecules and extending to the architecture of entire living systems. At the molecular level, chirality originates from asymmetric carbon atoms present in biomolecules such as amino acids and nucleotides. These stereogenic centers determine the three‐dimensional arrangement of substituent groups and establish the spatial characteristics of biological building blocks. As illustrated in Figure 2, l‐alanine and d‐alanine are enantiomeric forms that differ in the orientation of functional groups around the chiral carbon atom. Although they share the same molecular composition, their mirror‐image configurations generate distinct spatial arrangements that influence subsequent biological organization.

At the next level of hierarchy, the stereochemistry of these chiral monomers directs the formation of stereospecific polymers, including proteins and nucleic acids. The predominance of l‐amino acids in living organisms promotes the assembly of characteristic protein secondary structures, such as right‐handed α‐helices and defined β‐sheet conformations. Similarly, the stereochemical organization of nucleotides contributes to the structural integrity and handedness of DNA. These polymers subsequently fold into complex three‐dimensional structures that create catalytic active sites, ligand‐binding domains, and molecular recognition interfaces essential for biological function.

As shown in Figure 2, chirality is further propagated through higher‐order levels of biological organization. The stereospecific architecture of proteins and nucleic acids enables highly selective processes of molecular recognition and catalysis, which are fundamental to metabolic regulation, signal transduction, and genetic information processing. At the supramolecular and cellular levels, stereochemically compatible interactions govern the assembly and operation of ribosomes, polymerases, membrane‐associated complexes, and cytoskeletal structures. Collectively, this hierarchical progression from chiral molecules to stereospecific polymers, molecular recognition systems, and ultimately homochiral life ensures the fidelity, specificity, and efficiency of biological processes. Disruption of stereochemical compatibility can compromise structural stability, catalytic performance, and biological activity, highlighting chirality as a fundamental organizing principle of living systems (Sasabe and Suzuki 2018).

Recent theoretical analyses have further highlighted that chirality may propagate across multiple dimensional scales of organization. Molecular chirality influences helical handedness in one‐dimensional structures, which in turn shapes higher‐order folding geometries and supramolecular assemblies. This hierarchical propagation of stereochemical information reinforces the stability of biological systems and constrains the structural possibilities available during molecular evolution (Liu 2021). Consequently, the inversion of chirality in biological systems requires coordinated changes across multiple structural levels rather than modification of a single molecular component.

### 3.2 Stereochemical Incompatibility between Mirror and Natural Biomolecules

One of the central challenges in mirror biology arises from stereochemical incompatibility between molecules of opposite chirality. Molecular recognition in biological systems depends on highly specific spatial interactions between enzymes and substrates, nucleic acids and polymerases, or receptors and ligands. These interactions are governed by precise geometric alignment of chemical groups within binding interfaces. When molecules possess opposite chirality, this alignment cannot be achieved, resulting in reduced binding affinity or complete loss of recognition.

For example, natural proteases that evolved to recognize peptides composed of l‐amino acids exhibit very limited activity toward peptides composed of d‐amino acids. Similarly, polymerases that replicate natural nucleic acids cannot efficiently process mirror nucleic acids because the helical geometry and sugar stereochemistry differ fundamentally. This stereochemical mismatch disrupts hydrogen bonding patterns and catalytic positioning within enzyme active sites. As a result, mirror nucleic acids are typically resistant to degradation by natural nucleases, while mirror proteins exhibit resistance to many proteolytic enzymes (Harrison et al. 2023; Weinstock et al. 2014).

These observations demonstrate that mirror biological systems are inherently orthogonal to natural biological networks. While this orthogonality has been proposed as a potential mechanism for biological containment, it also represents a major barrier to constructing mirror organisms. To achieve a functional mirror cell, all interacting biomolecular components must share the same stereochemical configuration. Any mixture of natural and mirror components would compromise molecular recognition and disrupt cellular processes.

### 3.3 Structural Constraints in Mirror Nucleic Acid Systems

Nucleic acids occupy a central role in biological information storage and transmission. The stereochemistry of the sugar backbone determines the helical geometry of DNA and RNA molecules and influences their interactions with polymerases and regulatory proteins. Natural DNA adopts a right handed double helical structure that depends on the d‐configuration of its sugar backbone. When the stereochemistry of the sugar is inverted, the resulting nucleic acid adopts a mirror helical structure that is incompatible with natural replication and transcription machinery.

Experimental studies have demonstrated that mirror nucleic acids composed of l‐sugars can form stable double helices and participate in sequence‐specific base pairing. However, these mirror nucleic acids cannot be efficiently replicated or transcribed by natural enzymes because the catalytic interfaces of polymerases are stereochemically optimized for natural nucleic acids (Wang et al. 2016). The development of mirror polymerases capable of recognizing and processing l‐nucleic acids, therefore represents a major challenge in mirror biology.

In addition to polymerase recognition, nucleic acid chirality influences interactions with other cellular components such as ribosomes and regulatory proteins. The structural compatibility between nucleic acids and proteins is essential for gene expression and cellular regulation. Consequently, mirror organisms require a complete mirror genetic information system that includes mirror DNA, mirror RNA polymerases, mirror transcription factors, and mirror ribosomes.

### 3.4 Foldamer Perspectives and Stereochemical Constraints on Protein Structure

Insights from foldamer chemistry provide valuable perspectives on how stereochemistry constrains protein structure and function. Foldamers are synthetic oligomers designed to adopt well defined folded conformations based on the stereochemical properties of their monomer units. Studies in this field have demonstrated that small changes in monomer stereochemistry can dramatically alter folding patterns and structural stability (Gellman 1998).

These findings highlight the strong coupling between stereochemistry and protein folding landscapes. In natural proteins, the exclusive use of l‐amino acids restricts the conformational space available for secondary and tertiary structure formation. If proteins were constructed from d‐amino acids instead, the folding geometry would be mirrored but would still require consistent stereochemical organization throughout the polypeptide chain.

The alanine world model further illustrates how the selection of amino acid stereochemistry may have influenced early protein evolution. According to this model, specific amino acid stereochemistries shaped the structural possibilities of early peptides and contributed to the emergence of stable catalytic structures (Kubyshkin and Budisa 2019). These evolutionary considerations suggest that mirror proteins must follow analogous structural constraints to achieve stable folding and functional catalytic sites.

### 3.5 Implications for the Feasibility of Mirror Cellular Systems

The hierarchical nature of chirality imposes stringent requirements on the design of mirror biological systems (Table 1). Because biological processes rely on coordinated interactions among multiple stereochemically compatible components, the construction of mirror organisms requires reconstruction of an entire mirror biochemical infrastructure. This infrastructure includes mirror genetic information systems, mirror translation machinery, mirror metabolic enzymes, and mirror membrane biosynthesis pathways.

**Table 1:** Hierarchical levels of chirality in biological systems and implications for mirror life.

Level of organization	Natural biological configuration	Mirror biological configuration	Implications for mirror life
Molecular stereochemistry	l‐amino acids and d‐sugars	d‐amino acids and l‐sugars	Determines orientation of biomolecular building blocks
Secondary structure	Right handed alpha helices and natural beta sheet geometry	Mirror handed folding patterns	Requires consistent stereochemistry across proteins
Macromolecular folding	Natural protein tertiary structures	Mirror folded proteins	Catalytic sites must retain functional geometry
Nucleic acid structure	Natural DNA double helix	Mirror nucleic acid helix	Requires mirror polymerases for replication
Cellular networks	Integrated stereochemical compatibility	Mirror compatible molecular networks	Entire mirror central dogma required

Experimental advances in mirror protein synthesis and mirror nucleic acid chemistry demonstrate that individual components of such a system are chemically feasible (Harrison et al. 2023; Wang et al. 2016). However, integrating these components into a coherent cellular network remains an unresolved challenge. Each component must interact with others through stereochemically compatible interfaces, and small mismatches in geometry may compromise overall system functionality.

Consequently, mirror biology should be understood as a systems‐level engineering challenge rather than a simple molecular inversion problem. Future research will require the integration of chemical synthesis, structural biology, synthetic genomics, and systems engineering to reconstruct mirror cellular processes within a consistent stereochemical framework. By addressing these constraints, researchers may eventually determine whether mirror organisms represent a feasible extension of synthetic biology or remain primarily theoretical constructs.

## 4 Engineering Strategies For Mirror Biological Systems

The realization of mirror biological systems requires a coordinated engineering framework that reconstructs the essential processes of life within a stereochemically inverted molecular environment. Unlike conventional genetic engineering, which modifies existing biological systems, mirror biology aims to establish an entirely orthogonal biochemical architecture composed of mirror macromolecules and mirror metabolic networks. Achieving such systems demands the integration of advances in chemical synthesis, synthetic genomics, protein engineering, and orthogonal translation technologies. Several complementary strategies have therefore been proposed to guide the stepwise development of mirror biological systems.

### 4.1 Chemical Synthesis of Mirror Biomolecular Building Blocks

The first requirement for constructing mirror biological systems is the reliable synthesis of mirror biomolecular components (Table 2). Chemical methods have enabled the preparation of mirror proteins composed of d‐amino acids as well as mirror nucleic acids composed of l‐ribose sugars. These synthetic macromolecules serve as foundational components for mirror biochemical systems.

**Table 2:** Key technological strategies for engineering mirror biological systems.

Engineering strategy	Technological approach	Key challenges	References
Mirror macromolecule synthesis	Chemical synthesis of d‐proteins and L nucleic acids	Large scale synthesis and folding efficiency	Harrison et al. (2023); Weinstock et al. (2014)
Mirror replication systems	Engineering polymerases for mirror nucleic acids	Accurate replication of mirror genomes	Wang et al. (2016)
Mirror translation machinery	Ribosome engineering and orthogonal translation systems	Efficient incorporation of d‐amino acids	Katoh et al. (2017); Liu and Schultz (2010)
Synthetic genome construction	Chemical genome synthesis and genome rewriting	Stability and regulation of mirror genomes	Venetz et al. (2019); Lartigue et al. (2007)
Mirror metabolic pathways	Directed evolution and enzyme engineering	Compatibility with mirror substrates	Cobb et al. (2013a); Cobb et al. (2013b)
Orthogonal biological systems	Xenobiology and genetic firewalls	Isolation from natural biological networks	Schmidt (2010); Acevedo‐Rocha and Budisa (2011)

Native chemical ligation and related peptide synthesis approaches have been widely used to assemble d‐peptide chains and construct mirror proteins with defined stereochemistry. Experimental studies have demonstrated that d‐proteins can fold into stable three dimensional structures and retain catalytic activity that mirrors the function of their natural counterparts (Harrison et al. 2023; Weinstock et al. 2014). These findings confirm that stereochemicalinversion does not inherently prevent protein folding or enzymatic activity, provided that the entire polypeptide chain maintains consistent chirality.

Parallel developments in nucleic acid chemistry have enabled the synthesis of mirror DNA and RNA molecules. l‐nucleic acids exhibit strong resistance to degradation by natural nucleases because the stereochemical configuration of their sugar backbone prevents recognition by biological enzymes. Mirror nucleic acids therefore represent promising candidates for information storage and molecular recognition in synthetic biological systems (Wang et al. 2016). However, the efficient synthesis of large mirror nucleic acid sequences remains technically demanding and requires continued improvements in chemical synthesis technologies.

### 4.2 Development of Mirror Replication and Transcription Systems

The replication and expression of genetic information represent essential processes for any living system. In mirror biology, these processes require polymerases capable of recognizing and processing mirror nucleic acids. Natural DNA polymerases evolved to interact with nucleic acids containing d‐ribose sugars and therefore cannot efficiently replicate mirror nucleic acids.

Research in mirror molecular biology has demonstrated that mirror nucleic acid systems can support sequence‐specific base pairing and templated information transfer. Experimental models have shown that mirror genetic systems can participate in replication and transcription when supported by specially designed catalytic systems (Wang et al. 2016). However, developing mirror polymerases capable of copying long mirror genomes remains a major challenge.

Engineering such enzymes requires careful modification of catalytic domains to accommodate reversed stereochemical geometry. Structural insights into polymerase active sites suggest that stereochemical orientation of substrates plays a central role in nucleotide incorporation and catalytic fidelity. Consequently, mirror polymerases must be specifically engineered to recognize mirror nucleotides and maintain accurate information transfer during replication and transcription.

### 4.3 Engineering Mirror Translation Systems

Protein synthesis represents another fundamental barrier in the construction of mirror biological systems. Ribosomes in natural organisms are optimized to synthesize proteins composed of l‐amino acids and exhibit limited ability to incorporate d‐amino acids into polypeptide chains. This stereochemical restriction arises from the geometry of the ribosomal peptidyltransferase center and the interactions between transfer RNA molecules and the ribosome.

Experimental efforts to overcome this limitation have focused on modifying ribosomal structures and translation factors to improve the incorporation of d‐amino acids during protein synthesis. Engineering of aminoacyl‐tRNA synthetases has enabled the generation of aminoacyl‐tRNA substrates capable of participating in peptide bond formation under specific conditions (Katoh et al. 2017).

Orthogonal translation systems provide additional opportunities for developing mirror protein synthesis pathways. These systems utilize engineered transfer RNA and synthetase pairs that function independently from the native translation machinery, allowing the incorporation of non‐canonical‐amino acids into proteins (Liu and Schultz 2010; Wang et al. 2009). Such orthogonal frameworks may serve as transitional platforms for constructing translation systems capable of producing mirror proteins.

### 4.4 Synthetic Genome Design and Mirror Genomic Architecture

Synthetic genomics has transformed the ability of researchers to redesign genetic systems at the whole genome scale. Large scale genome synthesis projects have demonstrated that bacterial genomes can be chemically synthesized, assembled, and introduced into cells to produce viable organisms with modified genetic architectures.

The chemical rewriting of bacterial genomes has provided evidence that extensive genome recoding can be achieved while preserving biological functionality. These approaches involve systematic redesign of coding sequences, regulatory elements, and genomic organization to create programmable genetic systems (Venetz et al. 2019). Earlier studies in genome transplantation further showed that synthetic genomes can replace natural chromosomes and reprogram cellular identity (Lartigue et al. 2007).

Applying these strategies to mirror biology would require the construction of genomes composed entirely of mirror nucleotides. Mirror genomes must encode mirror proteins and interact with mirror transcription and translation systems. Designing such genomes also requires consideration of codon usage, regulatory sequences, and genome stability within a stereochemically consistent environment.

### 4.5 Engineering Mirror Metabolic Networks

For mirror biological systems to function as autonomous organisms, they must contain metabolic pathways capable of supporting energy production and biosynthesis. Natural metabolic pathways rely on enzymes that recognize substrates with specific stereochemical configurations. Mirror organisms would therefore require mirror enzymes capable of catalyzing biochemical reactions involving mirror substrates.

Protein engineering and directed evolution techniques offer potential strategies for optimizing mirror enzymes. Directed evolution has been widely used to modify enzyme specificity and catalytic efficiency by generating genetic diversity and selecting variants with improved performance (Cobb et al. 2013a; Cobb et al. 2013b). Applying these approaches to mirror enzyme design may allow the development of catalytic systems capable of sustaining mirror metabolic networks.

Another important consideration is the source of nutrients and metabolic substrates. Some metabolites used by biological systems are achiral and therefore could potentially be utilized by both natural and mirror organisms. However, many metabolic intermediates possess stereochemical configurations that may limit their compatibility with mirror enzymes. Designing metabolic pathways that rely on achiral substrates or mirror‐compatible intermediates therefore represents an important aspect of mirror biological engineering.

### 4.6 Integration of Mirror Cellular Systems

Although individual components of mirror biology have been demonstrated experimentally, integrating these components into a functional cellular system remains a major challenge. Biological systems rely on coordinated interactions among replication machinery, transcription systems, translation machinery, metabolic enzymes, and membrane structures. Each of these components must operate within a consistent stereochemical framework.

The concept of orthogonal biological systems provides a useful framework for addressing these challenges. Orthogonal systems are designed to function independently from natural biological networks and minimize cross interactions between engineered and natural components. Xenobiological strategies that incorporate alternative genetic codes or synthetic biochemical building blocks have been proposed as approaches for achieving such orthogonality (Acevedo‐Rocha and Budisa 2011; Schmidt 2010).

Recent developments in engineered microbial chassis and orthogonal translation systems demonstrate that synthetic biology can construct partially independent biological systems with reduced genetic compatibility with natural organisms. These advances may provide a foundation for future mirror biological systems that operate within an entirely separate stereochemical framework (Karbalaei‐Heidari and Budisa 2024).

### 4.7 Interdisciplinary Technological Integration

The development of mirror biological systems requires coordinated advances across multiple scientific disciplines. Chemical synthesis provides the tools needed to construct mirror biomolecules, while structural biology and protein engineering enable the redesign of enzymes and ribosomes. Synthetic genomics supports the construction of programmable genetic systems, and systems biology offers methods for integrating complex biological networks.

**Figure 3:**
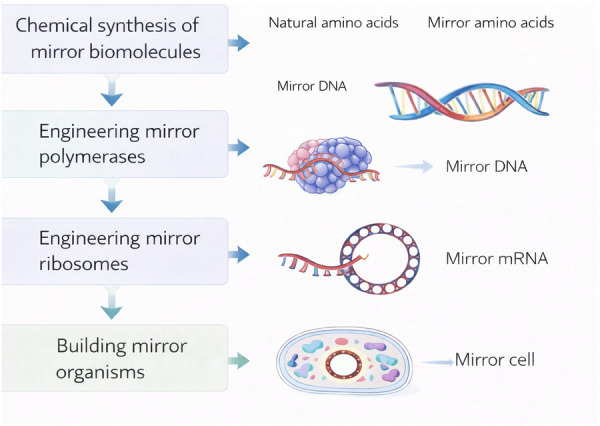
Engineering roadmap toward mirror biological systems.

Roadmap analyses of synthetic life construction emphasize the importance of interdisciplinary collaboration in achieving ambitious biological engineering goals (Figure 3). Progress toward mirror biological systems will therefore depend on coordinated efforts among chemists, molecular biologists, structural biologists, and computational scientists (Kriebisch et al. 2025). Such collaborative approaches are essential for addressing the complex challenges associated with reconstructing life within a mirror stereochemical framework.

## 5 Biosafety and Biocontainment of Mirror Organisms

**Figure 4:**
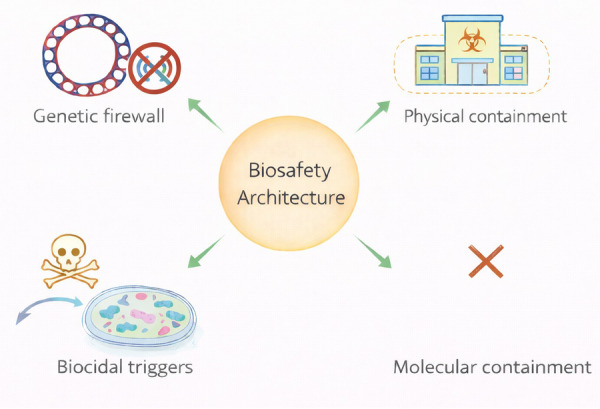
Biosafety architecture for mirror biological systems.

The potential development of mirror organisms raises important biosafety and biosecurity considerations that extend beyond conventional synthetic biology frameworks (Figure 4). Mirror biological systems are expected to exhibit stereochemical isolation from natural life because their molecular components possess opposite chirality. While this orthogonality may provide intrinsic containment advantages, it does not eliminate ecological or technological risks associated with engineered organisms. A comprehensive biosafety framework must therefore evaluate genetic containment, metabolic isolation, environmental interactions, and governance mechanisms that regulate the development and deployment of mirror biological systems.

### 5.1 Orthogonality and Intrinsic Biological Containment

One of the central arguments supporting mirror biological systems as a biosafety strategy arises from their biochemical orthogonality to natural organisms. Molecular recognition processes in living systems depend on stereochemical compatibility between enzymes, substrates, nucleic acids, and structural biomolecules. Because mirror organisms would operate with inverted molecular chirality, most natural enzymes and polymerases would be unable to process mirror biomolecules efficiently. Similarly, mirror proteins and nucleic acids would not be readily recognized by many biological components of natural ecosystems.

This stereochemical separation has been proposed as a mechanism that limits horizontal gene transfer and reduces the likelihood of genetic interaction between engineered mirror organisms and natural microbial communities. Xenobiology research has explored similar approaches through the development of alternative genetic systems designed to operate independently from natural DNA based biology (Schmidt 2010). The concept of a genetic firewall has also been proposed as a strategy to create organisms that are genetically isolated from natural biological systems, thereby reducing the risk of unintended gene flow (Acevedo‐Rocha and Budisa 2011).

Although stereochemical orthogonality may provide a degree of biological isolation, it should not be interpreted as complete biosafety. Biological systems interact with their environment through multiple chemical and ecological pathways, some of which may involve achiral metabolites or physical interactions that are not strictly dependent on stereochemistry. Consequently, mirror organisms could still influence ecological systems through resource competition, metabolite release, or other biochemical processes.

### 5.2 Synthetic Auxotrophy and Metabolic Containment

Synthetic auxotrophy represents one of the most widely discussed containment strategies in synthetic biology. In this approach engineered organisms are designed to depend on specific nutrients or chemical compounds that are not present in natural environments. This metabolic dependency prevents the organisms from surviving outside controlled laboratory or industrial settings.

In the context of mirror biology, synthetic auxotrophy could involve the requirement for mirror nutrients such as d‐amino acids or l‐nucleotides that are not naturally abundant in the environment. By restricting the availability of these essential components, the survival and replication of mirror organisms could be tightly controlled. Similar containment approaches have been explored in xenobiological systems that incorporate alternative genetic codes or synthetic metabolic pathways (Schmidt 2010).

Designing such metabolic dependencies requires careful consideration of nutrient uptake pathways, biosynthetic processes, and environmental availability of substrates. Metabolic engineering strategies can be used to eliminate endogenous biosynthesis pathways for essential molecules while introducing controlled supply mechanisms within laboratory environments. This approach provides an additional layer of containment that complements the inherent stereochemical isolation of mirror biological systems.

### 5.3 Genetic Firewalls and Orthogonal Translation Systems

Genetic firewalls represent an important biosafety strategy for engineered biological systems. These systems are designed to prevent genetic exchange between engineered organisms and natural populations by modifying fundamental processes of gene expression. One approach involves the use of orthogonal translation systems, in which engineered aminoacyl‐tRNA synthetases and corresponding tRNAs selectively recognize reassigned codons or noncanonical amino acids.

Orthogonal translation systems create a genetic code that differs from the universal genetic code used by natural organisms. As a result, genetic information transferred from engineered organisms to natural organisms cannot be properly interpreted, reducing the likelihood of functional gene transfer. Similarly, genetic material from natural organisms cannot be effectively expressed within the engineered organism (Acevedo‐Rocha and Budisa 2011).

Recent advances in synthetic biology have demonstrated the feasibility of integrating orthogonal translation systems into engineered microbial chassis. These systems provide enhanced control over gene expression and can be combined with synthetic metabolic pathways to create genetically isolated biological platforms (Karbalaei‐Heidari and Budisa 2024). Such strategies may be particularly valuable in mirror biology because they reinforce stereochemical isolation with genetic isolation mechanisms.

### 5.4 Ecological Interactions and Environmental Risk Assessment

Although mirror organisms may exhibit reduced compatibility with natural biological systems, their potential ecological impacts remain uncertain. Environmental interactions between organisms involve complex networks of chemical signaling, nutrient exchange, and resource competition. Some of these interactions may occur through molecules that do not possess strong stereochemical specificity.

A comprehensive risk assessment must therefore consider several ecological dimensions. One important factor involves competition for environmental resources such as carbon sources, nitrogen compounds, or micronutrients. Even if mirror organisms are not directly metabolized by natural microbes or predators, they may still influence ecological systems through resource utilization or production of metabolic byproducts.

Another concern involves the environmental persistence of mirror biomolecules. Because mirror proteins and mirror nucleic acids are resistant to many natural degradation pathways, they may accumulate in ecosystems if released unintentionally. The long‐term ecological consequences of such accumulation remain poorly understood and require further investigation (Adamala et al. 2024).

In addition, engineered organisms may produce secondary metabolites or signaling molecules that influence microbial communities. These interactions may occur even when stereochemical compatibility is limited. For this reason ecological risk assessment must examine not only genetic interactions but also chemical and metabolic effects on environmental systems.

### 5.5 Governance, Regulation, and Responsible Innovation

The development of mirror biological systems has prompted discussions regarding governance and responsible innovation in synthetic biology. Because mirror life represents a potentially transformative technological capability, regulatory frameworks must ensure that research proceeds under appropriate biosafety guidelines and ethical oversight.

International discussions have emphasized the need for transparency, risk assessment, and interdisciplinary collaboration when developing advanced synthetic biology technologies. Scientific communities have also called for careful evaluation of mirror biological research to ensure that potential benefits are balanced with environmental and societal considerations (Adamala et al. 2024).

Responsible innovation frameworks encourage the integration of biosafety research, ethical analysis, and regulatory planning throughout the research and development process. Such approaches help identify potential risks early and support the design of containment strategies that minimize environmental impact.

### 5.6 Future Directions in Biosafety Research

Further research is needed to develop robust biosafety frameworks for mirror biological systems (Table 3). Key priorities include the experimental validation of containment strategies, quantitative modeling of ecological interactions, and improved understanding of the environmental stability of mirror biomolecules.

**Table 3:** Biosafety strategies for containment of mirror biological systems.

Containment strategy	Mechanism	Advantages	References
Stereochemicalorthogonality	Mirror biomolecules incompatible with natural enzymes	Limits biochemical interaction with natural life	Schmidt (2010)
Synthetic auxotrophy	Dependence on synthetic nutrients absent in natural environments	Restricts survival outside controlled environments	Schmidt (2010)
Genetic firewall	Orthogonal genetic code prevents gene exchange	Reduces horizontal gene transfer	Acevedo‐Rocha and Budisa (2011)
Orthogonal translation systems	Engineered translation machinery interprets alternative codons	Enhances genetic isolation	Karbalaei‐Heidari and Budisa (2024)
Environmental risk assessment	Evaluation of ecological interactions and metabolite effects	Supports responsible deployment	Adamala et al. (2024)

Advances in systems biology, ecological modeling, and synthetic biology will provide valuable tools for evaluating the behavior of engineered biological systems in complex environments. These approaches can support the development of predictive models that estimate ecological risks and inform biosafety guidelines.

Ultimately, the safe development of mirror organisms will require a combination of technological safeguards, regulatory oversight, and interdisciplinary collaboration. By integrating stereochemical containment, metabolic dependencies, and genetic isolation mechanisms, synthetic biology may establish multiple layers of biosafety that support responsible innovation in mirror biological systems.

## 6 Ecological and Evolutionary Risks of Mirror Life

The prospect of constructing mirror organisms has stimulated significant discussion regarding their potential ecological and evolutionary consequences. Although mirror biological systems are expected to operate within a stereochemically isolated biochemical framework, this isolation does not necessarily eliminate all environmental risks. Ecological systems involve complex interactions among organisms, chemical compounds, and environmental resources. Consequently, the introduction of organisms that operate with inverted molecular chirality could generate ecological effects that extend beyond direct genetic interactions. Evaluating these risks requires careful examination of ecological compatibility, metabolic interactions, evolutionary adaptation, and environmental persistence.

### 6.1 Ecological Compatibility and Resource Competition

Ecological systems are structured around competition for limited environmental resources such as carbon sources, nitrogen compounds, and essential micronutrients. Even if mirror organisms are biochemically incompatible with natural organisms, they may still compete for shared environmental resources. Many nutrients available in natural ecosystems are achiral or possess chemical structures that may be utilized by both natural and mirror organisms. As a result, mirror microorganisms introduced into environmental settings could potentially compete with natural microbial populations for these resources.

Competition for ecological resources has the potential to alter microbial community structure and ecosystem function. Microbial communities regulate key environmental processes including nutrient cycling, organic matter decomposition, and primary productivity. If mirror organisms were capable of sustained growth in natural environments, their presence could influence the balance of microbial populations and potentially modify ecosystem dynamics. For this reason ecological compatibility must be evaluated not only in terms of molecular interactions but also in relation to resource utilization and metabolic capabilities (Adamala et al. 2024).

### 6.2 Resistance to Biological Degradation and Environmental Persistence

One characteristic frequently associated with mirror biomolecules is resistance to enzymatic degradation by natural organisms. Natural proteases and nucleases evolved to recognize biomolecules with specific stereochemical configurations. Because mirror proteins and mirror nucleic acids possess opposite chirality, these molecules are typically not efficiently degraded by natural enzymes. This resistance may confer increased stability to mirror biomolecules in environmental settings.

While enhanced molecular stability may be beneficial in biomedical or industrial applications, it also raises concerns regarding environmental persistence. If mirror organisms or their biomolecular products were released into ecosystems, they might remain intact for extended periods because natural degradation pathways may be less effective. Persistent biomolecules could accumulate in environmental compartments such as soil, sediments, or aquatic systems.

Long term accumulation of biologically active compounds may influence ecological processes by altering chemical signaling pathways or interacting with microbial communities. These potential outcomes remain largely unexplored and represent an important area for future research in environmental microbiology and ecological chemistry (Adamala et al. 2024).

### 6.3 Chemical Signaling and Microbial Communication

Microbial ecosystems rely on complex networks of chemical signaling molecules that regulate cooperative behavior, competition, and population dynamics. Processes such as quorum sensing depend on the production and detection of signaling molecules that coordinate gene expression across microbial populations. Many of these signaling systems involve molecules that interact with receptors through specific structural recognition mechanisms.

Mirror organisms may produce metabolites or signaling molecules that interact with natural microbial communities in unexpected ways. Some signaling compounds may be achiral or may possess limited stereochemical specificity, allowing them to interact with receptors in natural organisms. Alternatively, mirror metabolites could interfere with natural signaling pathways by acting as competitive inhibitors or by altering chemical gradients within microbial communities.

Understanding these interactions requires investigation of how mirror metabolites influence microbial communication networks and ecological feedback loops. Such studies will help clarify whether mirror organisms could disrupt natural microbial signaling systems or whether stereochemical differences would prevent meaningful interactions (Elder et al. 2020).

### 6.4 Evolutionary Adaptation and Long‐Term Dynamics

Evolutionary processes represent another important consideration in evaluating mirror biological systems. Evolution operates through mutation, selection, and genetic variation within populations. Over time organisms can adapt to environmental conditions and develop new biochemical capabilities. The possibility that mirror organisms could evolve in natural environments raises important questions regarding long‐term ecological dynamics.

One theoretical concern involves the possibility that mirror organisms could gradually evolve mechanisms to interact with natural biochemical systems. Although initial stereochemical incompatibility may limit such interactions, evolutionary processes could potentially produce enzymes or transport systems capable of recognizing both mirror and natural molecules. While such adaptations may be unlikely over short timescales, long term evolutionary processes may generate unexpected biochemical innovations.

Another consideration involves the stability of mirror genetic systems themselves. If mirror organisms rely on synthetic genetic components or engineered metabolic pathways, evolutionary pressures could favor mutations that restore compatibility with natural biochemical systems. Understanding these evolutionary trajectories will require experimental studies and computational modeling that examine mutation rates, selection pressures, and biochemical constraints in mirror biological systems.

### 6.5 Ecological Modeling and Risk Assessment

Because mirror biological systems have not yet been constructed as complete organisms, ecological risk assessment currently relies on theoretical modeling and extrapolation from related biological systems. Systems biology approaches and ecological modeling can help predict how engineered organisms might behave in complex environmental contexts.

Modeling frameworks can incorporate parameters such as growth rate, resource utilization efficiency, metabolite production, and environmental stability. These models allow researchers to explore potential scenarios in which mirror organisms interact with natural ecosystems and evaluate the consequences of different containment strategies. Integrating ecological modeling with laboratory experiments will improve the accuracy of risk predictions and guide the development of biosafety protocols.

In addition, environmental monitoring techniques can be used to detect the presence of engineered biomolecules and evaluate their stability in natural ecosystems. Such approaches provide valuable data for assessing environmental persistence and potential ecological impacts of synthetic biological systems.

### 6.6 Research Priorities for Ecological Risk Evaluation

Addressing ecological and evolutionary risks associated with mirror life requires coordinated research efforts across several scientific disciplines (Table 4). Environmental microbiology, ecological chemistry, systems biology, and synthetic biology all contribute important perspectives to the evaluation of engineered biological systems.

**Table 4:** Potential ecological and evolutionary risks associated with mirror biological systems.

Risk category	Description	Potential ecological consequences	References
Resource competition	Mirror organisms compete with natural microbes for nutrients	Alteration of microbial community structure and ecosystem processes	Adamala et al. (2024)
Environmental persistence	Mirror biomolecules resistant to natural enzymatic degradation	Accumulation of biomolecules in ecosystems	Adamala et al. (2024)
Chemical signaling interference	Mirror metabolites interact with microbial communication systems	Disruption of quorum sensing and ecological signaling	Elder et al. (2020)
Evolutionary adaptation	Mirror organisms evolve new biochemical capabilities	Potential emergence of unexpected metabolic interactions	Adamala et al. (2024)
Ecosystem level effects	Changes in nutrient cycling and microbial diversity	Long term ecological imbalance	Adamala et al. (2024)

Key research priorities include experimental studies on the environmental stability of mirror biomolecules, investigation of potential metabolic interactions with natural ecosystems, and development of predictive ecological models. In addition, interdisciplinary collaboration between scientists, regulators, and policy experts is necessary to ensure that mirror biological technologies are developed responsibly.

By integrating experimental research with ecological risk assessment frameworks, the scientific community can better understand the potential consequences of mirror biological systems and establish appropriate safeguards for their future development.

## 7 Future Perspectives in Chiral Synthetic Biology

Chiral synthetic biology has emerged as an interdisciplinary research frontier that integrates stereochemistry, molecular engineering, synthetic genomics, and systems biology. Although mirror organisms remain theoretical constructs, recent advances in mirror biomolecule synthesis, orthogonal translation systems, and genome engineering indicate that the fundamental principles required for mirror biological systems are gradually becoming experimentally accessible. Future progress in this field will depend on coordinated advances in molecular design, cellular engineering, ecological risk assessment, and regulatory frameworks. These developments will shape the direction of chiral synthetic biology and determine whether mirror biological systems can be safely implemented for scientific and technological applications.

### 7.1 Advancing Mirror Biomolecule Technologies

One of the most immediate research priorities in chiral synthetic biology is the improvement of methods for synthesizing mirror biomolecules (Table 5). Significant progress has been achieved in the chemical synthesis of mirror proteins composed of d‐amino acids and mirror nucleic acids composed of l‐sugars. Experimental studies have demonstrated that mirror proteins can adopt stable folded structures and perform catalytic functions similar to their natural counterparts (Harrison et al. 2023; Weinstock et al. 2014). Similarly, mirror nucleic acids have been shown to support sequence‐specific molecular recognition and information storage processes (Wang et al. 2016).

**Table 5:** Future research priorities in chiral synthetic biology.

Research area	Key objectives	Potential impact	References
Mirror biomolecule synthesis	Improve synthesis of d‐proteins and L nucleic acids	Enables experimental exploration of mirror systems	Harrison et al. (2023); Wang et al. (2016)
Orthogonal genetic systems	Expand genetic code and translation machinery	Supports development of alternative biological systems	Liu and Schultz (2010); Wang et al. (2009)
Synthetic genome engineering	Design programmable microbial genomes	Enables construction of customized biological platforms	Venetz et al. (2019); Lartigue et al. (2007)
Systems biology integration	Model interactions between engineered biological networks	Improves stability and efficiency of synthetic organisms	Solé et al. (2024); Jiang et al. (2025)
Mirror biomolecule applications	Develop therapeutic and diagnostic technologies	Expands biomedical and industrial applications	Lander et al. (2023); Qi et al. (2024)
Governance and biosafety	Establish regulatory frameworks for synthetic life	Ensures responsible innovation	Adamala et al. (2024)

Despite these advances, the large scale synthesis of mirror macromolecules remains technically challenging. Efficient production of long mirror polypeptides and mirror nucleic acid polymers requires improvements in peptide ligation techniques, automated chemical synthesis systems, and purification methods. Advances in asymmetric synthesis and structural design will also be essential for expanding the range of mirror biomolecules available for experimental research. Continued progress in these areas will provide the molecular components needed to explore mirror biological systems in greater depth.

### 7.2 Engineering Orthogonal Genetic Systems

Orthogonal genetic systems represent an important strategy for expanding the functional capabilities of synthetic biological systems. In these systems, engineered translational machinery operates independently of the natural genetic code used by conventional organisms. Orthogonal translation systems rely on engineered aminoacyl‐tRNA synthetase/tRNA pairs that selectively recognize reassigned codons and incorporate noncanonical amino acids into growing polypeptides.

Research on genetic code expansion has demonstrated that biological systems can incorporate a wide range of synthetic amino acids into proteins, thereby increasing the chemical diversity of cellular systems (Liu and Schultz 2010; Wang et al. 2009). These technologies provide valuable insights into how translation systems can be reprogrammed to accept unconventional molecular building blocks.

Future work in this area will focus on improving the efficiency and fidelity of orthogonal translation systems while minimizing cross interactions with native cellular machinery. Such systems may serve as transitional platforms for developing mirror translation systems capable of synthesizing proteins composed of d‐amino acids. In addition, orthogonal genetic systems may contribute to biosafety strategies by creating genetic codes that are incompatible with natural biological systems (Acevedo‐Rocha and Budisa 2011).

### 7.3 Synthetic Genome Engineering and Cellular Design

Advances in synthetic genomics have significantly expanded the capacity to redesign genetic systems at the whole‐genome scale. Large‐scale genome synthesis projects have demonstrated that bacterial genomes can be chemically assembled and modified to create organisms with redesigned genetic architectures. These technologies provide powerful tools for constructing programmable biological systems that can perform specialized functions.

The chemical synthesis and rewriting of bacterial genomes has shown that extensive modifications to genomic sequences can be implemented while maintaining cellular viability (Venetz et al. 2019). Earlier research on genome transplantation also demonstrated that synthetic genomes can replace natural chromosomes and determine the biological identity of recipient cells (Lartigue et al. 2007).

Future research will likely focus on applying these genome engineering techniques to create synthetic cellular platforms that support orthogonal biological processes. Such platforms may include engineered microbial chassis optimized for synthetic metabolism, alternative genetic codes, or non‐canonical biomolecules. These systems will provide experimental frameworks for exploring the feasibility of mirror biological architectures.

### 7.4 Systems Level Integration of Synthetic Biological Networks

Constructing complex synthetic biological systems requires the integration of multiple molecular processes including replication, transcription, translation, metabolism, and cellular regulation. Systems biology approaches provide valuable tools for modeling these interactions and predicting how engineered biological networks behave under different environmental conditions.

Computational modeling and network analysis can be used to simulate the dynamics of synthetic metabolic pathways and gene expression systems. These approaches enable researchers to identify potential bottlenecks, optimize pathway efficiency, and evaluate system stability. As synthetic biological systems become more complex, integrating experimental data with computational models will become increasingly important.

**Figure 5:**
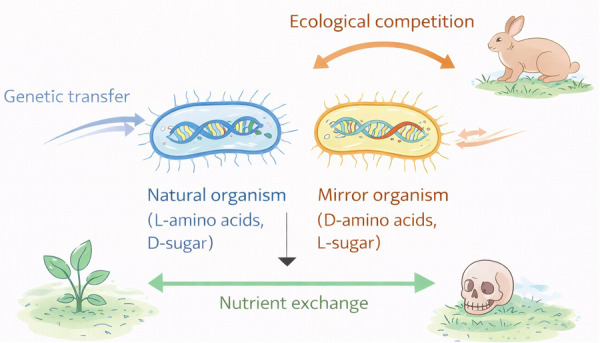
Potential ecological interactions of mirror organisms.

Recent studies on synthetic microbial ecosystems illustrate how engineered biological networks can be used to investigate ecological interactions and metabolic cooperation among microbial populations (Figure 5) (Solé et al. 2024; Jiang et al. 2025). Such research provides valuable insights into how synthetic biological systems may function in controlled environments and informs the design of more complex synthetic organisms.

### 7.5 Expanding Applications of Chiral Synthetic Biology

Beyond the development of mirror organisms, chiral synthetic biology has the potential to generate a wide range of applications in medicine, biotechnology, and materials science. Mirror proteins and peptides composed of d‐amino acids exhibit resistance to degradation by natural proteases, which enhances their stability in biological environments. These properties have stimulated interest in the development of d‐peptide therapeutics and mirror protein‐based drug discovery platforms (Lander et al. 2023; Qi et al. 2024).

Mirror biomolecules may also contribute to the design of highly stable diagnostic reagents and biosensors. For example, mirror protein scaffolds and mirror nucleic acid probes may exhibit improved resistance to enzymatic degradation in clinical samples. These properties could enhance the reliability and durability of diagnostic technologies.

In addition, mirror biomolecules may support industrial biotechnology applications that require stable catalytic systems. Mirror enzymes could potentially operate under conditions that degrade natural proteins, enabling new approaches to biocatalysis and chemical synthesis.

### 7.6 Governance and Responsible Innovation

As chiral synthetic biology continues to advance, responsible innovation will remain an essential component of research and technological development. Mirror biological systems raise complex ethical and regulatory questions regarding biosafety, environmental protection, and the governance of advanced biotechnology.

International scientific communities have emphasized the importance of careful risk evaluation and transparent communication when developing technologies that may alter fundamental biological systems (Adamala et al. 2024). Responsible research frameworks encourage collaboration among scientists, policymakers, and regulatory agencies to ensure that technological advances are implemented in ways that minimize potential risks.

Developing clear regulatory guidelines and biosafety standards will be essential for maintaining public trust and supporting sustainable innovation in synthetic biology. By integrating scientific progress with ethical oversight and environmental stewardship, the field of chiral synthetic biology can advance in a responsible and socially beneficial manner.

## 8 Conclusions

The concept of mirror biological systems represents a transformative frontier in synthetic biology that challenges long‐standing assumptions about the stereochemical organization of life. Natural biological systems exhibit a consistent pattern of homochirality in which proteins are composed of l‐amino acids and nucleic acids contain d‐sugars, enabling highly specific molecular recognition and coordinated cellular processes. Mirror biological systems propose a stereochemically inverted framework based on d‐amino acid proteins and l‐nucleic acids that would operate largely independently from the biochemical networks of natural organisms. Advances in chemical synthesis, protein engineering, and synthetic genomics have demonstrated that mirror biomolecules such as d‐proteins and l‐nucleic acids can form stable structures and perform fundamental biological functions, thereby providing experimental foundations for exploring mirror biological architectures. Nevertheless, the development of self‐replicating mirror organisms remains a complex challenge that requires the integration of mirror genetic systems, mirror polymerases, mirror ribosomes, and compatible metabolic pathways within a coherent cellular framework. In addition to technical barriers, mirror biological systems raise important questions concerning ecological interactions, evolutionary dynamics, and biosafety governance. Responsible advancement in this field will therefore require rigorous interdisciplinary collaboration among chemists, molecular biologists, systems scientists, and policy experts to ensure that emerging technologies are developed with careful consideration of environmental and ethical implications. Continued progress in chiral synthetic biology will deepen understanding of the stereochemical principles that govern biological organization while expanding the possibilities for innovative applications in biotechnology and medicine.

## Author Contributions

**Mohammad Nazrul Islam Bhuiyan:** conceptualization, writing – original draft, writing – review and editing, resources, visualization, validation.

## Funding

The author has nothing to report.

## Ethics Statement

The author has nothing to report.

## Conflicts of Interest

The author declares no conflicts of interest.

## Declaration of Generative AI and AI‐Assisted Technologies in the Writing Process

ChatGPT was employed solely to improve the clarity, grammar, and overall readability of the review paper. These tools were not used for content generation or idea development, and all original content was conceived and written by the author.

## Data Availability

Data sharing is not applicable to this article as no datasets were generated or analyzed during the current study.
